# Supplementary material for: Are carbon emissions trading and green financial instruments synergistic? -Comprehensive quantitative research based on content analysis
Source: PLoS One. 2024 Mar 7;19(3):e0298601. doi: 10.1371/journal.pone.0298601 (PMC10919601; doi:10.1371/journal.pone.0298601)
Supplement: S1 Data — (ZIP) [file pone.0298601.s001.zip › Data/地级市Z_F工作报告.zip!/╣π╢1⁄2/╔╟═╖╩╨/└┤╫╘╣1⁄2╓┌║┼í╛┬φ┐╦╩2╛▌═°í┐_2016.docx]

各位代表：

现在，我代表汕头市人民政府，向大会报告工作，请予审议，并请各位政协委员和其他列席人员提出意见。

一、“十二五”时期工作回顾

过去的五年，是汕头经济社会平稳较快发展的五年，是抢抓机遇、突破困境的五年，是夯实基础、蓄势跨越的五年。面对复杂多变的国内外经济形势，市政府在市委的正确领导和市人大、市政协的监督支持下，全面贯彻落实党的十八大、十八届三中、四中、五中全会和习近平总书记系列重要讲话精神，紧紧围绕国家“一带一路”重大战略，省委、省政府“三个定位、两个率先”目标和关于促进粤东西北地区振兴发展的决策部署，突出破解长期制约我市加快发展的瓶颈，促进经济社会发展迈上一个新台阶，完成“十二五”主要目标任务，为“十三五”时期振兴发展奠定坚实基础。

五年来，我们坚持科学发展理念，引领经济保持平稳较快增长。坚持稳中求进工作总基调，主动适应经济发展新常态，坚定不移实施投资拉动、消费推动、外向带动战略，统筹推进稳增长、调结构、促改革各项工作，促进经济提质增效，综合实力显著提升。2015年全市生产总值1850．01亿元，增长8．4％，五年间年均增长9．4％，高于全国、全省同期水平1．6和0．9个百分点，扭转了“十五”、“十一五”时期年均增速低于全国、全省的局面。人均生产总值33406元，年均增长8．4％。来源于我市的财政收入313．3亿元，年均增长12．9％；一般公共预算收入131．26亿元，年均增长12．6％；财政得益率从2010年的6％提高到7．1％，争取上级公共财政转移支付补助135．5亿元，是2010年的2．5倍。固定资产投资额1274．32亿元，增长27．1％，年均增长34．6％，“十二五”时期完成4108．6亿元，是“十一五”的3．2倍，长期以来投资率偏低制约经济发展的局面得到根本性改变。社会消费品零售总额1339．34亿元，增长12．9％，年均增长13．7％，规模列全省第五。外贸进出口总额92．85亿美元，是2010年的1．26倍，规模列粤东西北第一。港口货物吞吐量5180．9万吨，集装箱吞吐量117．9万标箱，分别比2010年增长47．6％、26．1％，汕头港进入世界港口集装箱吞吐量百强行列，成为国家“一带一路”战略中重点建设的15个港口之一。

五年来，我们坚持集中精力破解交通基础设施短板，对内对外互联成网的大交通骨架基本形成。我们深刻认识到交通滞后严重制约汕头的发展，坚持把交通基础设施建设作为重中之重，集中全市资源和力量，掀起前所未有的交通大建设热潮，交通建设取得历史性突破。“十二五”时期累计完成交通基础设施投资174．67亿元，是“十一五”的两倍多，在全省“交通基础设施提速升级指数”中列第一位，汕头作为区域交通航运中心的地位进一步巩固。境内总长163公里的5条高速公路建设同步推进，汕揭高速公路建成通车并与沈海高速互联互通，潮惠、揭惠高速公路均完成投资量超过五成，汕湛高速公路加快推进，潮汕环线高速公路先行工程开工建设。全市高速公路密度达3．7公里/百平方公里，在建高速公路建成后，高速公路密度将超过11公里/百平方公里，高于珠三角目前的水平。厦深铁路建成通车，使汕头与珠三角、厦漳泉形成两小时经济生活圈。人民群众热切期盼的广梅汕铁路增建二线及厦深铁路进汕头联络线工程动工建设并提前完成征拆，明年我们就能在市区乘坐动车。汕头疏港铁路完成预可研编制，粤东城际、市域轨道交通前期规划加快推进。城市候机楼一期工程建成投入使用，与潮汕机场实现无缝接驳。国道324线潮阳和潮南段、国道206线大学路段路面大修工程完成通车，连接广澳深水港与深汕、汕湛高速的疏港大道投入使用。“十二五”时期共完成国省道路面改造120公里，新农村公路建设568公里。南澳大桥建成通车，贯通汕头内海湾南北两岸的海湾隧道和横跨潮汕路、大学路的金凤路高架桥开工建设。广澳港区两个5万吨级泊位建成投产，两个10万吨级集装箱泊位和2万吨级石化码头动工建设，广澳防波堤工程完成投资量58％，华能海门煤炭中转基地完成起步工程。

五年来，我们坚持高起点规划拓展城市发展空间，大特区城市新格局初具规模。抓住经济特区范围扩大至全市域的契机，开展城市发展战略规划和城市总体规划修改，明确“精致汕头、潮人之都、滨海国际化山水人文都市”的城市发展定位，实施“北优、南拓、东扩、西联”战略，发挥放射状大交通骨架的先导作用，推动形成“一湾两岸”城市核心圈和“一核多组团”生态带形都市发展格局，中心城区首位度和区域中心城市集聚辐射、服务功能进一步提升。以“不求所有、但求所在”理念，引入战略投资者，开展城市重大战略平台合作。与中交集团合作开发东海岸新城，累计投资超过110亿元，全面完成围填海和水利主体工程建设，形成陆域面积20平方公里、海堤24公里，新津河大桥和外砂河大桥合龙，滨海大道等一批市政道路、管网及学校、医院等设施加快建设。与中国五矿集团合作开发粤东物流总部新城，土地一级开发和泰山路北延工程顺利推进。珠港新城粤东总部基地加快建设，认定总部企业73家，中国航天卫星大厦投入使用，国瑞会展酒店等首批企业总部大楼建设加快推进。与中信集团合作开发濠江新城，成为国内首个获立法保障的PPP项目，南滨片区累计投资超8亿元，潮汕历史文化博览中心主体结构建成，一批市政道路项目加快建设。依托中心城区空间拓展，谋划设立华侨经济文化合作试验区并获得国务院批准。试验区发展规划获省政府批准实施，基础设施、产业引进等专项规划编制完成，着力打造21世纪海上丝绸之路重要门户。潮阳东部新城、厦深高铁潮阳站（谷饶）物流新城等区域平台齐头并进，增强了城市副中心承载能力，城市可持续发展的空间和潜力进一步拓展，功能布局不断优化。

五年来，我们坚持强化产业引导扶持，产业转型升级呈现良好势头。大力实施“四大产业计划”和“五个100工程”，出台培育大型骨干企业、新一轮工业企业技术改造等政策措施，推动产业高端化。产业结构不断优化，三次产业比例由2010年的5．7：53．0：41．3调整为5．3：51．7：43．0。科技创新能力不断增强，新认定“中国驰名商标”15件、“广东省著名商标”74件、省名牌产品92个，新增高新技术企业35家，总数达到150家，列粤东西北首位；新增专利申请52983件、专利授权31908件、PCT国际专利申请239件，分别比“十一五”时期增长82．6％、83．1％、482．9％。金平区、澄海区被评为全国科技进步先进区，潮南区获批创建“全国服装产业知名品牌示范区”。促进传统产业改造提升，完成工业技改投资303．54亿元，是2010年的27．5倍。国家家用纺织品安全检测重点实验室、澄海省级出口玩具质量安全示范区通过验收。战略性新兴产业加快发展，广东移动粤东区域生产中心一期竣工，世纪互联大数据协同创新产业园启动建设，中国锆城核用条带、复合氧化锆、锆钛精选等项目竣工投产。大力发展现代服务业，苏宁广场、星湖城、长平新一城、卜蜂中心建成投入使用，宝奥城正大粤东配送中心开业，货运超市基本建成，华润万象城、百脑汇生活广场等一批商业综合体加快建设，粤东中心大都市现代化气息日渐浓厚。以电子商务、“互联网＋”为代表的新业态不断催生，全市近90％的企业开展电子商务应用，交易额突破900亿元，澄海玩具和潮南内衣家居服跻身“中国质造”首批上线的10个产业带行列，涌现出淘宝镇9个、淘宝村44个，数量分别列全国第一位、第四位，潮南区成为全国十大淘宝村集群之一。我市成为全国唯一同时获评“电子商务示范城市”、“信息消费试点城市”、“宽带中国示范城市”、“中国城市信息化50强”的地级市。邮政快递业持续加快发展，快递业务量和业务收入年均增长均超过50％，快递业务量列全省第六位、全国第35位。金融业稳健发展，全市金融机构本外币存贷款余额分别达到2857亿元和1199亿元，是2010年的1．5倍和1．8倍；新增银行、保险、证券等金融机构45家、小额贷款公司10家、融资担保公司8家；A股上市企业达到25家，列全省第三位，“新三板”挂牌企业25家，资本市场的汕头板块进一步壮大。文化创意产业持续高速发展，全市11家企业共投资超过60亿元进军动漫、游戏、影视等领域，奥飞动漫获评“十大最具影响力国家文化产业示范基地之一”。大力实施海洋旅游综合开发，南澳青澳海洋公园成为国家级海洋公园，莲花峰风景名胜区等4家景区成为AAAA级旅游区，莲华乡村旅游区获评“全国休闲农业与乡村旅游示范点”，丹樱生态园等一批新开发大型旅游区建成开放，喜来登、万豪等国际品牌酒店入驻汕头，区域旅游休闲中心凸显。农业渔业现代化迈上新台阶，澄海区成为国家现代农业示范区，新建省级现代农业“五位一体”示范基地3个，省级以上休闲农业与乡村旅游示范镇、示范点11个，县级农业龙头企业和农民专业合作社分别达85家和947家；建成海门、云澳2个国家级中心渔港。产业园区扩能增效取得新进展，省级工业园区产值超亿元企业118家，每平方公里创值33．05亿元。保税区获批设立B型保税物流中心，国家级高新区申报工作进展顺利。

五年来，我们坚持深化改革开放，发展动力和活力进一步增强。深入推进重点领域和关键环节改革，充分释放改革红利，全面激发社会活力，不断增强发展动力。商事登记实行注册资本认缴制、“先照后证”、“三证合一”等改革，营造宽松有序的准入环境，全市市场主体激增至24．4万户，注册资本1890．1亿元，分别比2010年增长56．4％和91．1％。不断简化企业投资管理流程，建立以备案制为主的企业投资项目管理体制。实施“营改增”税制改革，全面落实小微企业和高新技术企业等税收优惠政策，超过6万户小微企业享受免征增值税和所得税优惠。国企改革稳步推进，完成改革37家，化解债务6．84亿元，国企经济活力、控制力、抗风险能力不断增强。金融改革创新有新进展，通过盘活政府资源资产和优化投融资方式，化解政府性债务超过100亿元。汕头商行成功重组为广东华兴银行并通过债转股等方式消化政府债务近20亿元，2015年底华兴银行资产总额达到1062．11亿元，成立以来累计实现净利润4．94亿元，累计上缴各项税收6．22亿元。澄海农信社加快改制为农商行，汕特农信社改制工作扎实推进。设立全国首个以华侨为主题的金融股权交易市场“华侨板”，挂牌企业372家，累计意向融资额58．1亿元。文化体制改革取得突破，国有文艺院团改制组建成汕头市演艺集团有限公司，广东潮剧院、汕头潮乐团分别划转为潮剧研究传承中心、潮乐传承保护中心。华侨试验区获批试点设立港澳独资经营娱乐场所。推进民政工作改革创新，与国家民政部、省民政厅共建民政工作改革创新综合观察点，民政法制建设经验在全国推广。对外经贸合作活跃，五年累计实际吸收外商直接投资10．2亿美元，新设立境外投资企业66家，中方投资总额7．5亿美元。区域合作领域不断扩大，汕头大学与以色列理工学院合作创办广东以色列理工学院，规划建设中以（汕头）科技创新合作区，设立汕头中以国际技术转移中心，与以色列航空工业公司签署战略合作框架协议共建中以航空产业园，打造中以国家层面科教创新合作重大平台。华侨试验区被列入全国5个中欧区域政策合作试点地区之一，与西班牙安达卢西亚签订“一对一”结对合作协议。中国科协“海智计划”广东（汕头）工作基地、新侨创新创业基地、粤港澳服务贸易自由化省级示范基地等一批国家级、省级合作平台获批挂牌。建立汕潮揭同城化合作机制，在医保联网、年票互认、媒体互动、警务协作等方面取得实质性进展。

五年来，我们坚持统筹城乡发展，城乡人居环境明显改善。坚持城乡综合运营开发，加强投融资平台建设，成功发行城投债和企业债共计31亿元。建立健全土地储备经营机制，清理盘活闲置低效用地，“十二五”时期收储土地5372亩，供应土地面积33248亩，出让土地价款267亿元，土地价款是“十一五”时期的5．8倍，有效破解城乡建设资金瓶颈。持之以恒推进城市更新，城乡面貌明显改观。完成大学路、黄河路、长平路、中山路、长江路、外马路等破旧道路、水浸街和一批内街小巷路面改造，升级改造澄海、濠江、潮阳、潮南、南澳城区一大批市政道路。启动建设小公园开埠历史文化街区，实施首期永平路示范项目，外马路邮电广场和侨批文物馆新馆建成开放。完成一批“三旧”改造项目，西港、乌桥岛和光华片区旧城改造项目加快推进。创新城市管理体制机制，构建网格化、数字化管理模式，出台市级市政、园林维护量化标准，推行环卫、园林绿化、市政设施维护等作业层面市场化运作。深入开展城市管理联合执法，着力整治各种城市管理乱象。顺利通过国家卫生城市复检。加大生态环保力度，敢于直面解决城乡多年来累积的严峻生态问题。北轴、南区、清源等8座污水处理厂建成运营，粤东环境监控中心、澄海区洁源环保发电厂及新溪污水处理厂一期主体工程建成，市雷打石生活垃圾卫生填埋场扩建工程和环保电厂、潮阳和潮南两个垃圾焚烧发电厂、南澳垃圾无害化处理场动工建设。练江流域综合整治纳入省委、省政府重要议事日程，制定综合整治方案，完成环保、水利专项规划，省、市累计投入16亿元，启动潮阳、潮南两个纺织印染环保综合处理中心、陈店污水处理厂、练江干流陇田堤段堤防工程等一批环保水利基础设施建设。贵屿环境综合整治取得突破性进展，取缔、关停拆解户2469家，贵屿循环经济产业园区污水处理厂、电子废物集中拆解楼等建成投入使用，1461家拆解户组成49家企业全部入园实施统一管理，全面完成省督办验收项目建设，贵屿镇生态环境和村容村貌明显改善。加强水利基础设施建设，汕头大围六个应急堤段达标加固工程全面完成，潮阳引韩供水工程启动建设，“百河千沟万渠大整治”行动成效显著，集城乡水利防灾减灾、防洪治涝、灌溉供水于一体的现代化水利工程体系基本建成。全面开展“绿满家园”全民行动、“国家森林城市”创建活动、千村环境卫生整治行动和“美丽乡村、幸福村居”、名镇名村创建活动，建成国家级生态示范区1个、省级生态示范区33个，澄海区获评“美丽中国·最美城镇”，潮阳和舖、潮南东华、南澳后花园获评“中国美丽乡村”，潮阳区、南澳县成为省级新农村建设示范片。超额完成“十二五”节能减排目标。全面开展“平安汕头”建设和“三打两建”专项行动，大力开展“三类重点地区”整治，扎实推进“社会矛盾化解攻坚年”工作，全市刑事、治安警情、交通事故死亡人数逐年下降，人民群众安全感不断提升。不断加大食品药品监管力度，食品药品安全形势稳中向好。落实“党政同责、一岗双责、齐抓共管”机制，狠抓安全生产隐患排查治理，全市较大以上事故、各类事故死亡人数、亿元GDP死亡率分别比2010年下降100％、13％、44．4％，安全生产形势持续稳定好转，社会平安和谐。

五年来，我们坚持创新社会管理和公共服务，民生福祉持续改善。优先保障民生事业的公共财政支出，民生支出占公共财政支出比重达70％以上。累计投入37．51亿元，连续五年实施十件民生实事工程，民生实事群众知晓率列全省第六位。实施一系列更加积极的就业政策，五年来新增城镇就业35万人，农村劳动力转移就业28．7万人，就业形势保持稳定。城镇和农村常住居民人均可支配收入分别达23260．1元、12454．8元，年均增长11．6％和13．8％。全面推进教育创强，5个区（县）成为教育强区（县），69个镇（街道）全部成为教育强镇（街道）。持续促进教育公平，全面实施义务教育免试就近入学、优质高中指标到校和随迁子女在当地参加中考、高考等升学政策。大力改善办学条件，公办义务教育学校标准化覆盖率100％。教育质量继续提升，普通高考总上线率达92％，连续四年保持在九成以上。汕头大学综合实力不断提升，在泰晤士高等教育“2016金砖国家和新兴经济体大学排名”中列第160位，在入榜的大陆高校中列第33位。广东以色列理工学院获批筹设，一期校区启动建设。市高级技工学校成功申办技师学院。医疗服务能力明显提升，市中心医院门急诊综合楼、汕大医学院附一院和附二院住院综合楼、金平太安医院建成投入使用。五年新改扩建4个区县级医院、7个乡镇卫生院、4个社区卫生服务中心，新增三甲医院1所、三级医院6所，新增床位3234张、医疗服务场地23．68万平方米，医疗机构等级和医疗技术水平均居粤东榜首，区域性医疗服务中心辐射力不断增强。加强人口计生工作，稳定低生育水平，计生工作进位到省中等水平行列，金平区获评“全国阳光计生行动示范单位”。全民健身活动广泛开展，全市新增健身场地453个、面积143万平方米。社会保障制度基本实现全覆盖，在全省率先实行城乡一体化医疗保障制度，城乡居民医保人均补助标准由2010年的120元提高到380元，企业退休养老金从1059元提高到1868元，城乡居民基础养老金从55元提高到100元；在全省率先发行加载金融功能的社会保障卡，累计发卡520．3万张。加快保障性住房建设，筹集各类保障房2．07万套，新增发放租赁补贴4629户，全市保障性住房覆盖面达到20％。着力推进基础公共文化设施全覆盖，初步完成　“三馆一站”、村居（社区）文化室、公共电子阅览室的配套建设，基本形成市、区（县）、镇（街道）、村（社区）四级公共文化设施网络。市博物馆新馆正式开放。着力实施农村公益电影放映工程，完成多厅数字影院及广播电视“村村通”建设任务。加强非物质文化遗产保护，启动“海上丝绸之路”文物史迹申报世界文化遗产工作，市非物质文化遗产展示馆建成并免费开放，汕头抽纱、贵屿双忠信俗、陶瓷微书、太安堂中药文化等4个项目入选第四批国家级非物质文化遗产名录。侨批档案成功入选世界记忆名录，实现我市世界记忆文献遗产零的突破。加强文物保护工作，国民革命军东征军总指挥部、政治部旧址、文光塔、崎碌炮台被国务院列为全国重点文物保护单位。“南澳Ⅰ号”出水瓷器近3万件，水下文化遗产保护取得丰硕成果。加大公共交通投入，购置公交车959辆，新增公交线路40条，公交营运车辆和线路数量比“十一五”分别增长41％、53％，行政村客运通达率100％。大力推进一村（社区）一法律顾问工作，实现全市村居全覆盖。国防建设和民兵预备役工作得到加强，双拥工作深入推进，第五次获评“全国双拥模范城”。实施乡贤反哺工程，引导国内外潮商积极参与家乡经济和公益事业建设，设立省潮商公益基金会、潮阳公益基金会和潮南公益基金会，累计募集公益项目资金51．35亿元。全面完成第二轮扶贫开发“双到”工作，48个重点帮扶村和8个涉渔社区实现100％脱贫。建立健全精准助残制度，去年直接受益残疾人超过6万人次，获评“全国无障碍建设城市”、“全国白内障无障碍市”。全市殡葬惠民政策实现全覆盖。民族宗教领域和谐稳定。2015年确定的十件民生实事完成年度任务。

五年来，我们坚持打造阳光法治服务政府，政府作风改进和政务环境优化明显。自觉接受市人大及其常委会的监督和市政协的民主监督，办理人大议案14件、建议378件，政协提案836件。充分运用特区立法权和较大市立法权，提请市人大常委会审议法规草案23件，制定政府规章47件。在全国地级市率先出台行政程序规定、制度廉洁性评估办法等一系列政府规章，从制度层面规范行政行为。依法行政工作在全省2014年度依法行政考评中被评为优秀等次，金平区、濠江区获评“全国法治县（区）创建工作先进单位”。推进行政复议委员会试点工作，受理复议案件484宗。深化行政审批制度改革，共取消和调整市级行政审批事项495项，压减行政审批事项63．5％。全力推行政府工作部门权责清单制度，在全省率先同步公布市、区（县）政府工作部门权责清单目录。市县政府机构改革基本完成，在濠江区实行简政放权和大部制改革，获第二届“广东治理创新奖”行政改革奖。加快电子政务建设，全面启用“汕头政府在线”，全市134个单位上线实现办公自动化，各级4119项行政审批和便民服务事项进驻网上办事大厅，实现政务办公、行政审批、公众服务电子化；打造政府12345便民服务热线平台，有序推进公共资源交易体制改革和交易平台建设，形成一楼式集中服务、一站式网上服务、一号式语音服务、一体化公共资源交易服务“四位一体”的综合政务服务体系。汕头政府门户网站列全国政府网站绩效排行第19名、省第三名。市政府应急办官方微博列2015全国政务微博影响力排行第70名、省第10名。汕头企业服务微信公众平台获评2015年政府网站政务微信卓越奖。坚持落实监察、审计部门负责人固定列席市政府常务会议制度，全程介入政府决策的酝酿、讨论、决定、实施等环节，促进行政管理与行政监察、审计监督同步。深入开展党的群众路线教育实践活动、“三严三实”专题教育和“我服务我担当我阳光”进一步转变政府作风专项行动，严格落实中央“八项规定”，完成公务用车制度改革，大力整治公车私用、违规修建楼堂馆所、设立“小金库”和违规使用专项资金等行为，政府作风明显改进，政务环境不断优化。

各位代表！我市“十二五”时期改革发展成就来之不易，是省委、省政府和市委正确领导的结果，是全市人民同心协力、共同奋斗的结果，是海内外乡亲大力支持、积极参与的结果。在此，我代表市人民政府，向全市广大工人、农民、知识分子、干部职工，向给予我们支持的人大代表、政协委员、各民主党派、工商联、社会各界人士和海内外乡亲，向中直和省直驻汕单位，向驻汕解放军、武警官兵，向所有关心和支持汕头发展的港澳台同胞、海外侨胞和国际友人表示崇高的敬意和衷心的感谢！

但我们也清醒地认识到，与人民群众、海内外潮人的期盼相比，经济社会发展还存在很多亟待解决的问题：一是历史遗留问题和欠账多，加快发展和化解历史包袱的双重任务很重。二是经济持续加快增长的动力和后劲不足，特别是产业发展与土地、环境约束的矛盾突出，产业结构战略性调整任务艰巨，产业园区的承载能力和集聚作用还不强。三是城乡区域发展不协调，“半城半乡”问题突出，生态环境治理任务艰巨，城市管理亟待加强，交通秩序、市容市貌还没有根本性改观，群众意见较大。四是公共服务水平整体不高，教育、医疗、社会保障等民生事业发展滞后，与人民群众的期待仍有差距。五是建设阳光法治服务政府仍存在薄弱环节，干部创新意识、责任意识、担当意识、服务意识仍需加强。我们要正视这些问题，坚持目标导向与问题导向相统一，采取更加有力的措施，切实加以解决。

二、“十三五”时期的目标任务

根据《中共汕头市委关于制定国民经济和社会发展第十三个五年规划的建议》，我们编制了《汕头市国民经济和社会发展第十三个五年规划纲要》，提交大会审议。

“十三五”时期，是我市全面建成小康社会的决胜阶段，对汕头的发展至关重要。我们欣喜地看到，我市的发展基础不断夯实，发展态势不断向好，长期制约汕头发展的交通和环保基础设施等短板正在逐步补齐，一批事关汕头当前和长远发展的重大项目正在紧锣密鼓推进，特别是华侨试验区和中以（汕头）科技创新合作区等重大战略平台的加快建设，将为汕头未来发展提供新的空间和动力。尽管世界经济环境更为严峻复杂，国内经济发展中不平衡、不协调、不可持续问题仍很突出，区域竞争日趋激烈，我市在前进道路上仍面临诸多挑战，但只要我们坚定信心，不甘人后，锐意改革创新，奋力攻坚克难，就一定能够推进汕头振兴发展，就一定能够夺取全面建成小康社会新胜利。

今后五年，我市经济社会发展的主要奋斗目标是：到2020年，全市生产总值达到约3100亿元，年均增长9％左右，人均生产总值约5．5万元，年均增长8％左右，提前实现生产总值和城乡居民人均收入比2010年翻一番；地方财政一般预算收入达到176亿元，年均增长6％左右；固定资产投资年均递增18％，五年累计达1万亿元；产业结构更趋优化，研究与发展经费支出占地区生产总值比重达2％以上，形成一批有竞争力的战略性新兴产业和先进制造业产业集群，服务业增加值占地区生产总值比重超过48％；城乡居民收入增长和经济增长同步，城乡区域发展更趋协调，全市城镇化率达75％。

实现上述目标，全市上下必须按照“五位一体”总体布局和“四个全面”战略布局，牢固树立五大发展新理念，做到“五个突出”：突出创新发展，提高经济发展质量和效益；突出协调发展，着力优化城乡区域一体化发展格局；突出绿色发展，推进美丽幸福汕头建设；突出开放发展，加快构建全方位开放发展新格局；突出共享发展，让全市人民在共建共享发展中有更多获得感。

三、2016年工作安排

2016年是“十三五”规划开局之年，是率先全面建成小康社会决胜阶段的第一年，也是推进结构性改革的攻坚之年，做好今年的政府工作意义重大。今年经济社会发展的主要预期目标是：地区生产总值增长8．5％，固定资产投资额增长20％，进出口总额增长5％，社会消费品零售总额增长12％，地方一般公共预算收入增长6％，居民消费价格涨幅预期3％左右，居民人均可支配收入增长8．5％，城镇登记失业率控制在3％以内，节能减排降碳约束性指标完成省下达年度计划。

今年政府工作的总体要求是：全面贯彻党的十八届四中、五中全会精神，深入贯彻习近平总书记系列重要讲话精神，按照省委十一届五次、六次全会精神和市委十届六次全会的部署要求，适应和引领经济发展新常态，围绕结构深度调整、做大做强实体经济这一主线，实施供给侧结构性改革攻坚，提高经济发展质量和效益，为“十三五”率先全面建成小康社会开好局、起好步。

为实现上述目标，重点抓好以下工作：

（一）全力推进交通基础设施建设提速增效，加快建设粤东交通航运中心

围绕打造粤东交通航运中心，乘势而上打好交通基础设施建设攻坚战，确保交通基础设施建设提速增效。

港口方面。把港口建设作为全市“头号工程”，加快推进广澳港区二期工程两个10万吨级集装箱泊位、1个2万吨级石化码头、广澳港区防波堤、华能煤炭中转基地建设。完成广澳港区航道二期10万吨级航道工程项目前期工作，确保与10万吨级集装箱码头同步投产，启动广澳港区三期工程前期工作。推进榕江、濠江和韩江等航道整治升级。实施扶持汕头港集装箱运输发展政策，支持码头企业新开航线或加密航班，吸引腹地货源，促进港口货物吞吐量较快增长。强化广澳港区经济功能整合，建立保税、濠江、港口协调联动机制。优化港口通关环境，加快广澳港区口岸联检大楼建设，推进口岸“信息互换、监管互认、执法互助”和“单一窗口”建设。

高速公路方面。潮惠高速公路汕头段、揭惠高速陈沙公路至深汕高速段年底建成，汕湛高速公路、潮汕环线高速公路汕头段全面开工建设。

铁路（轨道交通）方面。全面推进广梅汕铁路增建二线汕头段及厦深铁路联络线工程、疏港铁路建设，加快市域轨道和粤东城际轨道交通项目前期论证。积极谋划漳州至汕尾段沿海高速客运专线，提前做好线位、站场等规划工作。推进汕头火车站综合交通枢纽项目前期工作，加快打造集立体式换乘中心、商贸会展、文化娱乐于一体的多功能、地标式新型城市综合体。

干线公路方面。加快省道337线濠江段30公里路面改造、潮南环城公路、磊口大桥续建、金凤路高架桥、大学路桥梁工程建设。开工建设省道336线南澳环岛公路路面大修工程。完成新农村公路110公里、县乡公路40公里改造。

（二）坚持以创新驱动产业转型升级，加快构建粤东现代产业高地

坚持创新发展理念，全面推动科技创新、产业创新、业态创新，加快打造粤东科教创新中心、商贸物流中心、金融服务中心，构建高端高质高新的现代产业体系。

大力提升创新驱动发展能力。发挥中以（汕头）科技创新合作区的引擎作用，深化中以科技创新合作，力争打造国家级创新平台。依托金平、龙湖等省级民营科技园及汕头软件园、专业镇建设，精准培育扶持一批科技型企业，争取新增50家高新技术企业。实施大中型企业研发机构全覆盖行动，培育扶持一批新型研发机构。实施科技企业孵化器倍增计划，依托高新区、汕头大学、广东以色列理工学院，引入社会资本建设一批国家级、省级的科技企业孵化器，鼓励发展众创、众包、众扶、众筹空间，加快构建完整孵化链条，争取新增3个市级以上科技企业孵化器。开展中小微企业创业创新城市示范基地工作，激发形成大众创业、万众创新热潮。

做大做强战略性新兴产业。重点发展新材料、大数据、通用航空等产业，推动产业向规模化、集聚化发展，形成新的支柱产业。加快东方锆业氧化锆项目和树业环保可降解环保材料产业基地项目建设。加快大数据协同创新产业园建设，吸引一批新一代信息技术企业落户。全力推动与以色列航空工业公司的战略合作，加快中以航空产业园建设，争取引入一批航空产业链项目。

加快发展现代服务业。大力发展现代商贸物流业，加快推进华润万象城、百脑汇生活广场等一批现代商贸综合体，苏宁易购粤东运营中心、京东粤东电商物流基地、国美物流园等现代物流园区，华侨创新产业城国际会展中心、珠港新城文化艺术交易中心等一批会展场馆建设。优先发展金融业，依托华侨试验区重点引进银行、证券、保险等各类金融机构的区域总部，培育新型金融机构，发展普惠金融和跨境金融；积极发展多层次资本市场，推动“华侨板”升级为广东华侨股权交易中心，争取新增一批上市企业。大力发展电子商务，加快柏亚电子商务产业园、EMS跨境电子商务园区、保税区跨境通微商创业园建设；培育扶持农村电子商务，推动形成更多的淘宝镇、淘宝村，打造一批农村电商品牌示范项目。加快推进快递下乡工程，发展跨境快递业务，构建更加完善的快递配送网络。实施旅游业转型升级和创新发展三年行动计划，加快南澳岛东海岸国际旅游度假区、潮人码头、礐石风景区水上欢乐世界建设，加强小公园老城历史文化街区旅游开发。大力发展文化创新设计、文化博览、动漫游戏等新兴产业，规划建设一批特色文化产业集聚区，培育一批领军型文化企业。

改造提升传统产业。全面实施工业转型升级三年行动计划，推进“十百千”工程，突出运用“互联网＋”，促进产业智能化、高端化。加快建设国家玩具质检中心、洗涤及护肤用品国家质检中心、省计量产业检测中心等公共技术服务平台，为传统产业改造升级提供技术支持。积极发展蓝色海洋产业，深入推进南澳国家级海洋生态文明示范区建设，加快南澳总兵府“海峡两岸交流基地”规划建设，扩大与福建、台湾地区海洋综合开发合作，打造粤闽台海洋经济合作圈核心区。

促进产业园区扩能增效。加快建设汕头国际云计算产业创新基地、金平现代产业集聚区、龙湖龙东产业园等一批专业特色产业园区。促进高新区与金平区优势互补，探索“政区合一”新体制，推动高新区升级为国家级高新区。加快省级现代农业示范区、潮南台湾农民创业园建设，大力扶持农业龙头企业、专业合作社、家庭农场等现代农业经营主体，培育生态农业、都市农业等新业态，促进农业现代化。

（三）扎实推进城市扩容提质，着力优化城乡区域协调发展格局

坚持协调发展理念，统筹做好城市规划、建设、管理工作，优化城镇体系空间布局，不断提高以人为核心的新型城镇化水平，推动区域协同、城乡一体。

加快华侨试验区建设。东海岸新城重点加快市政基础设施建设，建成滨海大道、新津片区8条市政道路和莱芜大桥等3座桥梁；加快建设华侨创新产业城，推动华侨贸易文化中心、华侨高新产业园、华侨健康园、华侨教育基地和华侨新村等一批现代产业项目落地。濠江新城重点启动汕头海湾隧道主体工程、南滨南路西段建设，推进潮汕历史文化博览中心和岸线景观改造工程建设。珠港新城重点加快建设雅士利、太安堂总部大楼和国瑞会展酒店，开工建设联泰、超声研究所总部大楼。

深化城乡综合运营开发。创新城乡发展投融资机制，大力推广PPP模式，面向社会资本招商，撬动和推进一批重大基础设施和产业项目建设。加快中以（汕头）科技创新合作区土地一级开发和金凤西路、金砂路西延、澄海路等路网基础设施建设。推进城市改造更新，加快嵩山北路、长江路、珠江路、华山路、红领巾路等一批破损道路、“水浸街”改造，打通庐山北路、龙江西路等一批“断头路”。建设海绵城市和地下综合管廊，修复城市水生态，涵养水资源，治理城市内涝。加快澄海、潮阳、潮南、南澳组团发展，推进潮阳东部新城、潮南行政新区、厦深高铁潮阳站（谷饶）物流新城等区域平台建设，分担城市功能，承接人口转移，增强城市副中心承载能力。支持濠江开展全省新型城镇化综合试点工作，在体制机制改革、“多规合一”等方面实现突破。推进节约集约用地及城乡土地生态利用制度综合改革试点工作，盘活闲置低效用地，提高土地保障水平。加快智慧城市建设，推进城市基础设施智能化，着重发展第四代移动通信，推进城市百兆光纤工程和宽带乡村工程，推进宽带提速普及和WiFi热点广覆盖。

提升城市管理水平。积极回应市民集中反映的“脏、乱、差、堵”等“城市病”，开展城市管理“大整治、大提升、大改观”专项整治行动，努力营造整洁、有序、畅通的人居环境。加大市容环境整治力度，推进街区形象提升工程、小贩疏导工程、集贸市场升级改造工程、病媒生物防制工程，加强禁摆道路管理，实施环卫管理网格化、市场化、一体化，着力解决城市重点区域　“牛皮癣”、占道经营、“黑烧烤”、违法搭建等问题。加速数字化城市管理建设，提升城市管理信息化、科学化水平。系统梳理城市交通拥堵顽疾，优化交通流组织设计，实施主干道快速路改造，全面提升道路通行能力和通行效率。实施公共交通优先发展战略，结合快速路网改造，探索设置公交专用道，调整优化公交线网布局，加快建设智能调度管理系统，提升市民公交出行的便捷性。推进道路交通秩序整治，重点查处摩托车乱闯红灯、不按道行驶等交通违法行为，取缔无牌无证、假牌假证、报废摩托车，有序设置摩托车禁行区域，努力实现道路交通秩序明显改观。

加强城乡生态环境建设。推进森林进城、绿道绕城、活水环城，争创“国家森林城市”和“国家生态园林城市”。建设城市中央公园、红树湾公园、北郊公园，实施牛田洋生态绿地建设工程，推进龙湖沟、黄厝围沟、西港河等水质提升工程。加快新津河“一河两岸”规划建设，打造生态和休闲相融合的绿色宜居带。加强环保基础设施建设，开展西区污水处理厂前期工作，推进新溪污水处理厂及配套管网、雷打石生活垃圾卫生填埋场扩建工程、雷打石环保电厂、中心城区污泥集中处置项目和潮阳、潮南区生活垃圾焚烧发电厂建设。

巩固贵屿环境污染整治成果，制定贵屿电子废物拆解行业污染防治管理办法，推进贵屿循环经济产业园区建设，实施严格圈区管理，推行清洁生产，杜绝污染反弹。持之以恒推进练江流域综合整治，加快纺织印染环保综合处理中心和铜盂、和平、陈店、司马浦、陇田等污水处理厂及配套管网建设，全面推行治污“河长制”，扭转练江水质恶化趋势。加强水利基础设施建设，全面实施练江流域综合整治防洪排涝水利工程，推进“千里海堤”、病险水库、水闸除险加固。开展国家节水型城市创建工作，推进南澳、潮阳、潮南引韩供水和“村村通自来水”工程，确保城乡安全供水。扎实推进“美丽乡村、幸福村居”创建活动，加快新农村示范片建设工作，启动“十镇百村”省级卫生镇村创建活动，积极创建宜居社区、村庄、城镇。

（四）以华侨试验区建设为引领，全面提升改革开放水平

坚持开放发展理念，以华侨试验区建设引领重点领域和关键环节改革，以改革促进开放，以开放倒逼改革，全面提升开放型经济发展水平。

推进供给侧结构性改革。制定“去产能、去库存、去杠杆、降成本、补短板”行动方案，完善差别化产业政策，在做优增量、调整存量、主动减量上精准发力。盘活国有资产存量，全面推动优质骨干国有企业改革。开展降本增效专项行动，落实减税降费政策，清理行政审批中介服务费，加快电力、流通市场化改革，降低企业生产要素成本。通过加快农民工市民化、发展住房租赁市场等，打通供需渠道，防范房地产库存风险。探索公共产品和服务供给新方式，着力提高供给体系质量和效益。

深化行政管理体制改革。建立华侨试验区决策委员会、发展咨询委员会、管理委员会“三位一体”管理体制，行使市一级和部分省级经济社会管理权限。加强权责清单管理，推行行政审批标准化建设，强化事中事后监管，规范行政权力运行。深化公共资源交易体制改革，形成规则统一、公开透明、服务高效、监督规范的公共资源交易平台体系。探索行政执法体制改革，加大城市管理、食品药品、工商质监等领域执法资源整合力度，推行综合执法。深化事业单位分类改革，加大行业事业单位优化整合力度，增强事业单位活力。

完善现代市场体系。加快华侨试验区政策创新，复制推广自贸区改革创新经验，重点推进金融、教育、文化、医疗等领域有序开放，争取国家和省在跨境金融、资源能源交易、文化创意等方面率先给予更多的试验权。深化商事制度改革，探索推进全程电子化登记管理方式。加快市场主体信用信息公示平台建设，构建以企业信息公示、信用监管为核心的市场监管新机制。

构筑开放合作新格局。加强以东盟为重点的国际合作，将华侨试验区打造成为中国—东盟自贸区升级版的门户和前沿。积极申办“海上丝绸之路论坛”、“中国侨商大会”等全球性大会，筹建世界华裔青年联合会，在华侨试验区设立永久性会址。组织编制中欧区域合作试点工作规划，规划建设中欧合作示范中心，推进建设一批重点合作项目。发挥中国科协“海智计划”广东（汕头）工作基地作用，招引海外智力人才。着力发展外贸新业态，大力培育外贸综合服务企业发展，支持跨境电商园区和公共海外仓建设，支持旅游购物出口，争取开展市场采购贸易试点。建成保税物流中心（B型）并投入使用。深化粤港澳服务贸易合作。制订“走出去”战略行动计划，出台境外营销网络建设和海外并购扶持措施，支持企业开展境外品牌、技术和产能并购，带动技术、产品、服务输出。

（五）统筹推进基本公共服务均等化，着力增强人民群众的获得感和幸福感

坚持共享发展理念，以更大的投入和更实的举措，加快发展教育、文化、卫生、体育等社会事业，提高城乡基本公共服务优质化、均等化水平，让人民群众在共建共享发展中有更多获得感。

提高就业和收入水平。完善创业扶持政策，鼓励以创业带动就业。探索“互联网＋创业孵化基地”模式，建立面向大众的创业服务平台。构建新型和谐稳定劳动关系，维护职工和企业合法权益。新增城镇就业5万人，农村劳动力技能培训2．55万人，农村劳动力转移就业4万人。实施城乡居民收入倍增计划，稳步调整企业职工最低工资标准，建立健全工资正常增长机制，缩小城乡、区域、行业收入分配差距。

推动教育均衡优质发展。启动全市教育现代化建设工作，实施初中学校品质提升三年行动计划，开展创建省一级学校、国家级示范性普通高中活动，继续推进教育信息化建设与应用，促进基础教育高水平均衡发展。做好潮阳、潮南教育强区、义务教育发展基本均衡区及全市教育强市验收工作。继续实施学前教育第二期三年行动计划，实现全市学前教育毛入园率90％以上、残疾儿童毛入园率75％以上。推进中等职业教育布局结构调整，加快鮀滨职业学校新校区和卫生学校异地重建步伐，办好汕头职业技术学院和汕头市技师学院，支持金平职校与吉林大学珠海学院建设广东城市建设大学。全力推进广东以色列理工学院建设并争取招生，支持汕头大学建设高水平大学，支持粤东高级技工学校办学。继续实施特殊教育提升计划，加快澄海、潮阳、潮南特殊教育学校建设。实施学校体育三年行动计划和中小学校园足球计划，增强学生体质。推动市电大业大职大向开放性大学转型，加快建立终身教育服务体系。支持和规范民办教育发展。加强师德师风建设，推动区域教师交流轮岗，鼓励高校毕业生到农村任教。

完善多层次社会保障体系。继续实施城乡居民大病医疗保险制度，鼓励发展补充医疗保险和商业健康保险。加快发展养老服务业，继续实施普惠型高龄津贴制度和银龄安康行动。支持社会力量举办养老机构，推广邻里互助养老新模式。全面落实推进机关事业单位工作人员养老保险制度并轨工作。试点开展普惠型儿童福利制度建设，提升儿童福利水平。扩大社会福利普惠面，促进社会慈善事业发展，完善社会救助体系。

推进健康汕头建设。深化医药卫生体制改革，完善多层次医疗卫生服务体系，着力建设卫生强市。加快推进汕大医学院附属肿瘤医院、市中医院、市妇幼保健院、市皮肤医院、汕大国际眼科中心异地建设，启动建设白求恩潮阳医院、国瑞医院、东方医院和仁济骨科医院，支持汕大医学院建成粤东医教、科研和民众健康教育推广中心，支持市二医院筹建粤东应急医院。重视急性传染病及慢性非传染性疾病的防治，加快规划建设传染病院。推进城市公立医院改革和分级诊疗体系建设，改进医保支付方式，破除以药补医模式。开展镇卫生院标准化建设，提升县级医疗机构服务能力。建设覆盖全市医疗卫生机构的区域医疗卫生信息平台和统一标准的居民电子健康档案，促进医疗卫生服务和健康信息的共享利用。积极推动中医药事业发展，大力发展健康服务业，促进医养结合。深化人口计生综合改革，健全计划生育服务管理体系，全面实施一对夫妇可生育两个孩子政策。深化殡葬改革，推广绿色生态殡葬。

实施精准扶贫攻坚工程。以农民人均可支配收入4000元为扶贫标准，将3．25万相对贫困户、10．56万相对贫困人口作为帮扶对象，重点帮扶37个相对贫困村。实施精准扶贫、精准脱贫，规划到户、责任到人，采取产业扶持、技能扶持、转移就业、生态补偿、低保兜底等方式，提高扶贫开发的精准度和有效性。

加快文化体育事业发展。推进市美术馆、档案馆新馆、侨批文物馆、侨批记忆广场等文化设施建设。改造升级“农家书屋”，完成市、区（县）图书馆“一证通”项目建设。推进“海上丝绸之路”申遗工作和非物质文化遗产传承体系建设，启动创建国家级潮汕文化生态保护实验区工作。加强城市文化形象推介，打造城市文化品牌。加快构建全民健身公共服务体系，完善城乡社区体育健身设施，广泛开展全民健身活动。

维护社会平安和谐。继续深化“平安汕头”创建，完善立体化治安防控体系建设，健全维稳应急处突工作机制，不断提高人民群众安全感。抓好综治信访维稳，加强社会矛盾排查化解。全面启动“七五”普法。健全“党政同责、一岗双责、齐抓共管”工作机制，严格执行安全事故预警防控和约谈制度，强化预防治本，保持全市安全生产形势持续稳定好转。健全完善企业信用监管，扎实推进诚信市场和平安市场创建工作。完善市、县、镇三级食品药品安全监管网络，加快安全信用体系和追溯体系建设，推进家禽“集中屠宰、冷链配送、生鲜上市”工作，保障“舌尖上的安全”。促进社会治理精细化，加强社区管理服务，激发社会组织活力。完善农村集体“三资”管理服务平台建设，夯实农村基层民主自治基础。推动军民融合发展，做好国防动员、“双拥”、优抚安置等工作，巩固军政军民团结。

继续办好十件民生实事。市级财政将投入11．5亿元，集中力量办好十件民生实事。一是巩固提升底线民生保障水平，二是促进教育资源公平均衡配置，三是改善城乡医疗卫生服务，四是加大对困难弱势群体帮扶力度，五是实施污染治理和生态建设工程，六是加大公共交通投入和交通综合整治，七是实施文化惠民工程，八是强化低收入住房困难群体的住房保障，九是继续实施“绿满家园”全民行动，十是改善农村生产生活环境。

（六）深入推进阳光法治服务政府建设，加快营造国际化法治化营商环境

以特区立法为引领，以加强电子政务建设为重要抓手，全面推进依法行政，进一步打造阳光法治服务政府，为汕头加快振兴发展营造国际化法治化营商环境。

切实加强民主法治建设。自觉接受市人大及其常委会的法律监督、工作监督和市政协的民主监督，认真听取人大代表、政协委员、民主党派、工商联、无党派人士和各人民团体的意见，切实提高人大代表建议和政协提案办理质量。

深入推进依法行政。坚持用好特区立法权，健全立法与重点领域和重点环节改革的有效衔接。有序推进立法协商工作，研究立法项目征求意见新机制，探索立法专家咨询制度，提高立法质量。推进重大行政决策程序立法和合法性审查，增强法律审查在政府重大决策中的风险防范功能。健全政府法律顾问制度。落实《汕头市行政程序规定》，规范行政执法、行政听证、行政公开等程序。

加强电子政务建设。加快推进区县政务外网覆盖建设，打造全市统一的电子政务网络平台。稳步推进市网上办事分厅建设，加快网厅进一步向基层延伸，打造省、市、区（县）、镇（街）、村（社区）五级网上办事服务体系。建设“网上12345热线服务平台”，推进政府语音服务向网上延伸。推广应用网上办事大厅手机版、企业专属网页和市民网页。

坚持不懈抓好政府作风建设。严格落实中央八项规定，巩固拓展党的群众路线教育实践活动成果，推进“三严三实”专题教育制度化，深入开展“我服务我担当我阳光”进一步转变政府作风专项行动，完善作风建设长效机制。实施阳光政务，全面深化政务公开。严格落实党风廉政建设主体责任制，完善监察、司法、审计等联动的行政权力监督机制，推进廉洁政府建设。抓好政府系统党风廉政教育和公务员队伍建设，促进干部清正、政府清廉、政治清明。

各位代表，过去五年，我们一道栉风沐雨、砥砺前行，为汕头加快振兴发展打下坚实基础。未来五年的蓝图已经绘就，美好前景令人鼓舞。让我们紧密地团结在以习近平同志为总书记的党中央周围，在省委、省政府和市委的坚强领导下，团结带领全市人民，凝聚海内外潮人的智慧和力量，乘势而上，奋力拼搏，为率先全面建成小康社会而努力奋斗！
